# Dyadic Coping and Its Underlying Neuroendocrine Mechanisms – Implications for Stress Regulation

**DOI:** 10.3389/fpsyg.2018.02600

**Published:** 2019-01-09

**Authors:** Anna-Lena Zietlow, Monika Eckstein, Cristóbal Hernández, Nora Nonnenmacher, Corinna Reck, Marcel Schaer, Guy Bodenmann, Markus Heinrichs, Beate Ditzen

**Affiliations:** ^1^Center for Psychosocial Medicine, Institute of Medical Psychology, Heidelberg University Hospital, Heidelberg, Germany; ^2^School of Psychology, Pontifical Catholic University of Chile, Santiago, Chile; ^3^Center for Psychosocial Medicine, General Psychiatry, Heidelberg University Hospital, Heidelberg, Germany; ^4^Department of Psychology, Ludwig-Maximilians-University, Munich, Germany; ^5^School of Applied Psychology, ZHAW Zurich University of Applied Sciences, Zurich, Switzerland; ^6^Department of Clinical Psychology, Children, Youth and Family, Institute for Psychology, University of Zurich, Zurich, Switzerland; ^7^Laboratory for Biological and Personality Psychology, Department of Psychology, Albert-Ludwigs-University of Freiburg, Freiburg im Breisgau, Germany

**Keywords:** dyadic coping, couple conflict, oxytocin, HPA-axis, cortisol, relationship satisfaction

## Abstract

Previous research suggests that neuroendocrine mechanisms underlie inter-individual stress coping in couples. The neuropeptide oxytocin (OT), while regulating stress-sensitive HPA-axis activity might be crucial in this process. The purpose of this study was to examine the impact of dyadic coping abilities and OT on HPA-axis outcomes and constructive behavior during couple conflict. We conducted a secondary analysis of our previous database (Ditzen et al., 2009), assessing the modulating role of dyadic coping and intranasal OT on couple conflict behavior. The data revealed a significant interaction effect of the dyadic coping by oneself score and OT on cortisol responses during couple conflict, suggesting that particularly individuals with low *a priori* dyadic coping benefit from OT in terms of dampened HPA-activity. The results are in line with previous research suggesting OT’s central role for stress regulation and prosocial behavior. Furthermore, an interaction with dyadic coping indicates adaptations in the sensitivity of the OT system during the individual attachment and relationship history. These data add to the evidence that the neuroendocrine attachment systems influence couple behavior. Future studies of neurobiological mechanisms underlying dyadic coping will be of high relevance for the development of prevention and intervention programs.

## Introduction

Conflicts *per se* are no indicator for a dysfunctional relationship, as they form part of social cohabitation. The way a couple manages to solve problems and conflicts in a constructive way and deals with negative affect are of vital importance not only for couple satisfaction but also for stress reaction and health which is also shown on neurobiological level: Research regarding couple interaction has shown that couples differ in the extent and the frequency of positive and negative affect and their psychobiological reactions toward a conflict situation (Ditzen et al., 2013; Baucom et al., 2015). The neuromodulator oxytocin (OT) seems to be involved in the rewarding and stimulating aspects of social interaction on a central nervous system level (Insel and Young, 2001; Zietlow et al., 2016; Feldman, 2017). Above this, OT has a regulatory effect on neuroendocrine stress responses (Neumann, 2002; Sullivan and Dufresne, 2006; Eckstein and Hurlemann, 2013), thereby modulating cognitive and endocrine regulation of stress and affect (Heatherton and Wagner, 2011).

In a secondary analysis of our database (c.f. Ditzen et al., 2009, 2013) we examined the links between OT, dyadic coping and cortisol reactivity and behavior during couple conflict interaction in a sample of healthy couples. Prior to the conflict discussion, couples self-administered intranasal OT or placebo, so that the effects of external OT in interaction with dyadic coping style could be investigated.

## Background

### Dyadic Coping

The theoretical framework of the Systemic Transactional Model (Bodenmann, 1995, 2005a) proposes that in a committed close relationship the daily stress experiences of one partner concern both partners, either directly (e.g., both partners are affected by the same stressor or the couple relationship causes stress) or indirectly (one partner’s stress spills over to the couple relationship) representing a dyadic phenomenon (Randall and Bodenmann, 2009). Therefore, dyadic coping which is defined as the couples’ mutual, interpersonal stress regulation and the dyadic capacity to deal with couple external stressors, has become a central concept within couple research (Bodenmann, 1997, 2000, 2005b). Coping dyadically with stress always includes both stress expression and dyadic support (Bodenmann, 2005a).

Functional and flexible emotion and stress regulation capacities of the partners are of particular importance in this process (Rusu et al., 2018) which in turn lead to increasing relationship satisfaction (Bodenmann et al., 2006; Herzberg, 2013; Falconier et al., 2015) and improved stress recovery (Ditzen et al., 2008, 2011; Meuwly et al., 2012). During the transition to parenthood these processes are even more important as they affect not only the couple but also the forming family with the risk to spill over to the next generation and hence to affect child socio-emotional development.

### Neuroendocrine Mechanisms Involved in Coping With Stress

#### HPA Axis Activation

Neuroendocrine regulation of stress is a central aspect of mental and physical health with the hypothalamic-pituitary-adrenocortical (HPA) axis as one major regulating system to cope with stress on a hormonal level (Sapolsky et al., 2000). HPA axis responses are mediated through a cascade of hormones from the central nervous system (corticotrophin releasing factor, CRF) which then stimulate adrenocorticotropic hormone (ACTH) and cortisol-secretion in the periphery. Based on the dynamic negative feedback of the HPA axis at every level, the increase of cortisol will– via activation of mineralocorticoid and glucocorticoid receptors– reduce further activation and initiate recovery from stress (McEwen, 2000). Cortisol, as the end-product of HPA activation, can be assessed from saliva, which is frequently done, in order to monitor endocrine stress-responses (Allen et al., 2017; Goodman et al., 2017).

#### Oxytocin

The neuropeptide OT has been known for centuries in gynecology for its role in giving birth and breastfeeding. In recent decades, the focus has changed toward the central effects on social cognition and stress regulation. Synthesized in the paraventricular nucleus of the hypothalamus, OT is released from the pituitary and reaches central and peripheral action sites. The central key nodes are situated mostly within the amygdala, insula and prefrontal areas, highly relevant for socio-emotional cognition, and top-down control of emotion, perception and behavior (Meyer-Lindenberg et al., 2011), e.g., in the context of fear (Eckstein et al., 2015). Human studies from our own group using intranasal application of OT have shown its involvement in social behavior, a.o. constructive behavior and stress-regulation during couple conflict interaction both via the HPA and sympathetic nervous system activation (Ditzen et al., 2009, 2013). On the other hand, OT release seems to be triggered by social contact and intimacy (de Jong et al., 2015), and therefore was hypothesized to build a self-reinforcing system. Importantly, OT seems to modulate these saliency and stress-regulatory functions from infancy on (Doom et al., 2017).

While early studies assumed these stress dampening and anxiolytic effects are evident in general (Kirsch et al., 2005; de Oliveira et al., 2012), newer studies showed that OT effects depend on the context (Olff et al., 2013). e.g., stress dampening effects become especially evident when combined with social support by a close person (Heinrichs et al., 2003; Eckstein et al., 2014). Above this, it has been suggested that OT does not have a prosocial or stress dampening effect in general, but depending on individual traits and experiences (for an overview see Olff et al., 2013). One underlying mechanism that is discussed to define this difference is OT receptor density or sensitivity in specific brain areas (Ross et al., 2009). OT-receptors, however, cannot be investigated in the human living brain, so far. Therefore, research designs rely on intranasal administration of OT in combination with fine-grained self-report and behavioral paradigms, in order to capture specific OT-functioning during social interaction and its effects on stress responses. Such studies suggest that the activation of central OT mechanisms in a social context might influence perceptions of salience and, thereby, moderate behavioral and physiological responses to social stimuli.

Another modulatory factor is gender. There are recent studies reporting gender-dimorphic effects of OT in various domains, including attachment behavior (Scheele et al., 2012; Preckel et al., 2014) and social cognition (Hoge et al., 2014; Gao et al., 2016). These results suggest that naturally occurring higher levels of estrogens in females and testosterone in males might be involved in the sex-specific behavioral and physiological effects (Ditzen et al., 2012) in response to stressors (Taylor et al., 2000). Gao et al. (2016) hypothesized that these sex-dimorphic effects have evolved from different roles for male and female humans in parenting, raising offspring and protecting the family. The modulatory role of gender on couple behavior is well documented. Early studies by Gottman (1994) and Gottman et al. (1998) have suggested that men rather react to conflict with avoidance and women with persistence. Newer studies have expanded this notion to differential responses on HPA activity (Ditzen et al., 2012; Laurent et al., 2013).

### Neuroendocrine Mechanisms of Attachment

Neuroendocrine theories on attachment and bonding propose a refined interplay of central nervous neuropeptides (OT), neurotransmitters (dopamine) and also steroids (e.g., cortisol) in the regulation of attachment bonds throughout the life span, both for parental attachment and pair bonding (Feldman, 2017; Frisch et al., 2017). OT seems to be involved in the rewarding and stimulating aspects of both adult dyadic interaction and parent’s emotional bonding to their infant (Insel and Young, 2001).

While parent-child attachment is motivated by caregiving, and characterized by asymmetric communication, the adult pair bond is motivated by the need for symmetric communication, and emotional and physical intimacy. However, both kinds of relationships involve the need for support and emotion/stress regulation within in the dyad.

### The Present Study

It seems plausible that not only individual traits and experiences, but *inter*-individual/ social characteristics impact the effects of OT on stress reactivity as well as interaction behavior. For the relevant social relationships in adulthood, primarily the intimate couple relationship, this would be experiences with dyadic stress coping. So far there are no investigations on the interplay of dyadic coping, as a couple’s style to deal with stress, and OT’s effects on physiological and behavioral stress responses.

Therefore, in a secondary analysis of our previous database (Ditzen et al., 2009) we were interested to examine the links between intranasal application of OT, reported dyadic coping, cortisol reactivity, and positive behavior during couple conflict. While we have shown before (Ditzen et al., 2009) that OT has beneficial main effects, the presented new analyses take new factors and outcomes into account in order to disentangle whether individual’s dyadic coping influence these effects. Based on the above cited studies on the individual traits modulating OT effects, we expected that dyadic coping abilities would modulate the effects of OT during dyadic interaction. Precisely, we assumed that both OT and positive dyadic coping would improve behavior and reduce cortisol responses to instructed couple conflict in the laboratory. In an exploratory hypothesis, we were also interested in the interaction of these two factors, thereby investigating whether either those couples with high or low dyadic coping would particularly benefit from OT application.

## Materials and Methods

### Participants

Forty-seven heterosexual couples (*n* = 94 subjects), aged 20–50 years, who were married or had been cohabiting for at least 1 year participated in the study. One couple (*N* = 2 subjects, OT group) missed to complete the Dyadic Coping Inventory and was therefore excluded from the present analysis. Exclusion criteria for participation were smoking, chronic mental or physical illness, medication intake and, for women, the intake of hormonal contraceptives, current pregnancy, and breastfeeding. All women were investigated during the luteal phase of their menstrual cycle. Subjects were informed that we were interested in hormonal influences on couple communication and that they would receive either OT or placebo before a conflict conversation in the laboratory. All couples gave written informed consent and were offered 100 Swiss Francs for participation. The Dyadic Coping Inventory (Bodenmann, 2008b), the General Health Questionnaire (GHQ; Goldberg and Williams, 1969), the Relationship Questionnaire (PFB; Hahlweg, 1996), and the Short Chronic Stress Scale (SSCS; Schulz et al., 2004) were presented to all study participants before the lab appointment, in order to assess trait characteristics. All participants provided written informed consent prior to participation. The study was approved by the ethics committee of the University of Zurich and the Canton of Zurich.

### Procedures

Experiments took place in the laboratories of the Department of Psychology at the University of Zurich. To control for diurnal variation in salivary cortisol, all assessments took place between 5:00 and 7:30 pm. After baseline salivary cortisol assessment and a pregnancy test in women, subjects rated the intensity of 23 pre-determined areas of couple conflict (Hahlweg, 1996) with regard to their own relationship. Couples chose two topics (e.g., finances, educational issues, leisure time) of continuing disagreement for the later discussion (Gottman, 1994; Weiss and Heyman, 1997; Kaiser et al., 1998). After this procedure, in a double-blind design based on the randomization table prepared by the study pharmacy, couples self-administered either 40 IU (5 puffs in each nostril) of OT (Syntocinon Spray, Novartis, Basel, Switzerland) or placebo (containing all ingredients except the active ingredient OT) intranasally under the supervision of the study coordinator. Forty-five minutes after drug administration, couples were asked to discuss the conflict issue that they had chosen previously during the following 10 min (Fehm-Wolfsdorf et al., 1999). Couples were alone in the room and were videotaped during this conflict discussion. After the conflict discussion, all subjects were asked to evaluate the discussion with a standard evaluation questionnaire (Hahlweg and Jacobson, 1984) on self-perceived aspects of the conflict (e.g., validity of the task, stressfulness of the task) and subsequently watched a nature movie for relaxation. Salivary free cortisol was repeatedly assessed with Salivette collection devices (Sarstedt, Sevelen, Switzerland) at baseline (-50 min), immediately before conflict (-1) and after conflict (+10, +20, +35, and +55 min). Saliva samples were stored at -20°C until required for analysis with a commercially available chemiluminescence immunoassay (CLIA; IBL Hamburg, Germany) with inter- and intra-assay coefficients of variation below 10%. Salivary cortisol levels were interpreted on the basis of the area under the curve with respect to ground (AUCg) after the conflict discussion, which allows a sensitive measure of physiological changes over time (Pruessner et al., 2003).

### Couple Conflict Behavior

Conflict behavior was coded with an adapted version of the Specific Affect Coding System (SPAFF; Gottman and Krokoff, 1989; Gottman, 1994) and the Coding System for Marital and Family Interaction (KPI; Hahlweg et al., 1984) with a computer-aided system of analysis [Computer Aided Observation System (CAOS); Bourquard et al., 2005]. Two trained raters who were blind with regard to the subjects’ group assignment coded non-verbal (e.g., eye contact, non-verbal positive behavior, and non-verbal negative behavior) and verbal behavior (e.g., curiosity/care, emotional self-disclosure, agreement, contempt, belligerence, and defensiveness). Inter-rater reliability (Cohen’s kappa) was 0.66 for non-verbal categories and 0.80–1.0 for verbal categories. Before calculating the sum scores, all behavior categories were z-transformed to make them comparable.

To consider both positive and negative categories of interactional behavior, a ratio was calculated dividing the score of total positive behaviors by the scores of total negative behaviors. This was achieved by adding a constant of 3 to both scores before the calculation of the ratio to transform them into positive values. By doing this, when the ratio was above 1, a higher duration of positive than negative behaviors was interpreted, while when the ratio was less than 1 the opposite could be concluded. Mean values, standard deviations, and ranges of the variables can be seen in Table 1.

**Table 1:** Descriptive statistics of dyadic coping, cortisol concentration, and behavioral ratio.

		Range	Minimum	Maximum	Mean	S.E.
Female	Total DCI	48.00	82.00	130.00	105.830	1.491
	Self DCI	24.00	43.00	70.00	56.700	0.819
	Partner DCI	26.00	19.00	45.00	36.511	0.744
	Cortisol	307.00	53.85	360.85	158.693	10.144
	Behav.Ratio	1.40	0.56	1.96	1.050	0.037
Male	Total DCI	59.00	68.00	127.00	102.477	1.631
	Self DCI	24.00	43.00	67.00	54.057	0.853
	Partner DCI	31.00	14.00	45.00	35.640	0.836
	Cortisol	552.83	40.50	593.33	184.254	14.403
	Behav.Ratio	1.17	0.50	1.67	1.011	0.339

*Total DCI, Dyadic Coping; Self DCI, Dyadic coping by oneself; Partner DCI, Dyadic coping by the partner; Cortisol, Cortisol AUCg; Behav. Ratio, Positive/Negative Behavioral Ratio, S.E., Standard Error.*

### Dyadic Coping

Dyadic coping was assessed with the Dyadic Coping Inventory (DCI; Bodenmann, 2008b). It assesses one’s own stress communication (“What I do when I am stressed?”) and that enacted by the partner (“What does my partner do when he/she is stressed?”). Each partner’s view of how they cope as a couple (common dyadic coping) is evaluated (“What we do when we are stressed as a couple?”). Hence, each single individual receives scores, rather than a joint score for the couple. The DCI contains 37 items which are rated on a 5-point scale (“0 = never” to “4 = very often”). In this study we used the total score “DCI total without evaluation,” which is the established total score of the inventory (Bodenmann, 2008a) and the scales “Dyadic Coping by the Partner” and “Dyadic Coping by Oneself.” The latter scale measures the participants self-evaluation of how they support their partners to cope with the conflict. This refers to emotional support and informational forms of support, which do not relieve the partner of the coping work, but rather support coping efforts, e.g., empathic understanding or assistance in the analysis of the problems (informational support). Higher scores indicate more supportive dyadic coping strategies. The DCI is a validated instrument in western cultures as well as in China (Xu et al., 2016).

### Statistical Analyses

Statistical Package for the Social Sciences (IBM^TM^ SPSS^®^ v. 24.0) was used for the descriptive analyses and plots. An Intra-Class correlation coefficient of ICC = 0.257 for cortisol AUCg and ICC = 0.502 for the positive/negative behavior ratio indicated non-independence of the data. Given the data is nested on dyads, and to test for group differences regarding HPA-axis reactivity after couple conflict in the treatment and placebo group, we conducted a hierarchical linear dyadic analysis following Kenny et al. (2006) recommendations for the adjustment of standard errors. For the hierarchical models, the library nlme (Pinheiro et al., 2018) of the statistical environment R (R Core Team, 2017) was used. A two-level hierarchical linear model was fitted using a Restricted Maximum Likelihood (REML) estimation method, because it offers robust estimates for small samples (Peugh, 2010). The intercept was set at random to allow for an adjusted estimation of standard errors by the upper level, while no random slopes were calculated given the limitations of having only two units per couple. All predictors were grand-mean centered, while dichotomous variables were effect coded to help with the interpretation of the results (Kenny et al., 2006). Models were visually evaluated for normality and equality of variance of the residuals. Because a pattern of increased dispersion of residuals by the fitted values was found for cortisol analyses, every model was weighted by them using the “power of the covariate” function of the nlme package (Pinheiro and Bates, 2000). A likelihood ratio test was conducted to evaluate improvement in model fitness. Equal variances were assumed for dyad members in every model because no improvement in model fit was found when set as different. Aggregated cortisol levels (AUCg, for details see Pruessner et al., 2003) were added as the dependent variable and group (OT vs. placebo), dyadic coping (total score) and their interaction were computed as predictors controlling for sex, age, and body mass index. To disentangle the confounded self and partner effect of the total DCI, secondary analyses were conducted using the Dyadic Coping by Oneself and Dyadic Coping by the Partner Scale. Afterward, a fourth model was conducted to determine differences in the ratio of positive/negative behavior duration between group and Dyadic Coping interaction, including the same control variables.

## Results

### Oxytocin, Dyadic Coping and HPA-Axis Reactivity to Couple Conflict

**FIGURE 1:**
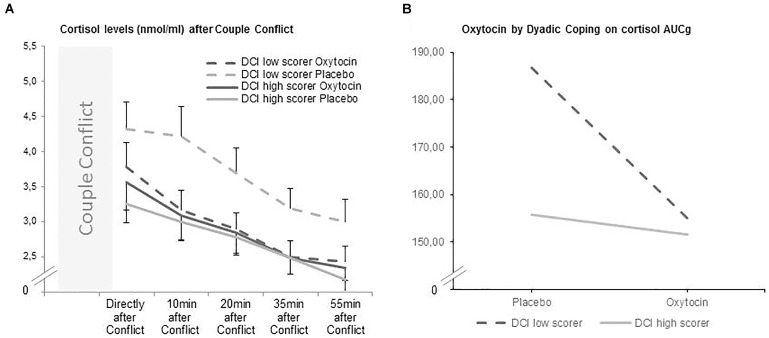
**(A,B)** Oxytocin significantly decreased the level of salivary cortisol (AUCg) after the couple conflict in individuals with low scores of DCI total. DCI low scorers are marked with a continuous line, while DCI high scorers are marked with a dashed line.

For the total DCI score, the likelihood ratio test showed an improvement in model fit when adjusted for the heteroskedastic pattern of residuals [L.Ratio (1) = 11.837, *p* < 0.001]. A trend interaction effect was found between group and dyadic coping (β = 1.392, *t*(33) = 1.958, *p* = 0.059) on cortisol levels after the conflict discussion (see Table 2). This trend effect is depicted in Figure 1A. When the dyadic coping by oneself was entered into the equation, the likelihood ratio test also showed an improvement in model fit when adjusted for the increase on residual dispersion by predicted values [L.Ratio (1) = 10.846, *p* = 0.001]. A significant interaction effect between self-dyadic coping [β = 2.966, *t*(33) = 2.190, *p* = 0.036] and OT on cortisol values was found (Table 2). Finally, when dyadic coping by the partner was entered into the equation, the likelihood ratio test also showed an improvement in model fitness when adjusted [L.Ratio (1) = 8.382, *p* = 0.004], however, no interaction effect was found between the partner’s coping [β = 2.681, *t*(33) = 1.731, *p* = 0.091] and oxytocin on personal cortisol values (Table 2).

**Table 2:** Dyadic modeling of Cortisol on oxytocin and dyadic coping.

	Total DCI	Self DCI	Partner DCI
**Fixed effects**			
	168.826		168.395
(Intercept)	(7.984)^∗∗∗^	169.019 (8.274)^∗∗∗^	(8.161)^∗∗∗^
Group	-6.045 (7.937)	-5.905 (8.202)	-6.058 (8.201)
DCI	-1.402 (0.705)	-2.018 (1.347)	-1.826 (1.540)
Age	-0.444 (1.348)	-0.337 (1.424)	-0.714 (1.454)
BMI	-5.406 (2.619)^∗^	-6.351 (2.730)^∗^	-4.523 (2.891)
Sex	10.538 (7.689)	11.007 (8.181)	11.252 (8.140)
Group^∗^DCI	1.392 (0.711)	2.966 (1.354)^∗^	2.681 (1.549)
**Random effects**			
Intercept	0.021	0.025	0.029
Residual	0.034	0.034	0.044

*^∗∗∗^p < 0.001, ^∗∗^p < 0.01, and ^∗^p < 0.05; Unstandardized beta coefficients are presented with their standard errors in parentheses. Dependent variable is AUCg of cortisol after the couple’s conflict.*

To further inspect the significant interaction effect of dyadic coping by oneself with OT in cortisol levels, a median split of the DCI scores into high and low values showed that specially individuals scoring low in self-dyadic coping benefitted from OT in terms of dampened cortisol levels (see Figure 1B). The general scale showed the same substantive pattern of results.

### Oxytocin, Dyadic Coping and Behavior Ratio During Couple Conflict

No significant interaction effect was found for the total DCI [β = -0.003, *t*(32) = -1.282, *p* = 0.209], dyadic coping by oneself [β = -0.003, *t*(32) = -0.663, *p* = 0.512], or by the partner [β = -0.008, *t*(32) = -1.673, *p* = 0.104]. The same was the case for the main effects. Interestingly, however, on a descriptive level the data showed the same pattern as for the cortisol results where participants with low DCI (median split) showed more positive than negative behaviors in the OT group, while they showed more negative than positive behaviors in the placebo condition. On the other hand, participants with high DCI showed more positive behaviors in both conditions. Analyses of the self-evaluation questionnaire with similar models did not yield any significant results.

## Discussion

In this presented secondary analysis (for former analyses of the dataset see also Ditzen et al., 2009, 2012) we were interested in the neuroendocrine aspects of dyadic stress coping traits and their specific influence on stress responses during couple conflict. With the administration of OT, a neuropeptide hormone associated to improved social attachment and reduced stress reactivity we aimed at imitating endogenous neural processes and tested the interactive effect of OT with couples’ self-reported dyadic coping.

Results showed a significant interaction of dyadic coping and OT on cortisol responses to instructed couple conflict and indicated that particularly those participants with low *a priori* scores of dyadic coping benefitted from OT in terms of dampened HPA-axis. As an extension of our former analyses, we could show that improvements on the group level (Ditzen et al., 2009) where mostly driven by individuals with least coping skills.

### Physiological Stress Responses

On the one hand, the cortisol results are in line with the broad range of literature showing effects of OT in down-regulating the HPA-axis, such as our previous analyses of the data (Ditzen et al., 2009). In accordance, e.g., Cardoso et al. (2013) demonstrated that OT attenuated the cortisol response elicited by physical stress. However, there are also studies that failed to find an effect of OT on cortisol levels (de Oliveira et al., 2012).

Above this, our significant interaction of OT with dyadic coping is in line with previous reports about person-dependent effects of OT (Olff et al., 2013), which suggest that personal traits moderate the effects of OT. In the present study, especially individuals who reported low levels in dyadic coping benefitted from OT treatment in terms of decreased cortisol levels. Interestingly, this effect was driven by the subscale “Dyadic Coping by Oneself.” The total DCI scale is composed both by the perceived coping skills of the individual and, at the same time, the perception that the individual has on the coping skills of his/her partner. However, because the interest of this study is on the individual effects, the perception of the partners coping skill would confound both the self and partner coping skills within the interaction effect and render it uninterpretable in meaningful terms. Therefore, it’s plausible that the subscale “Dyadic Coping by Oneself” turned the effect. Indeed, this adds further emphasis to the moderating role of the individual person factors. Those participants who stated that they fail to support their partners in their stress, showed most stress-dampening effects of OT. This might be due to their deficits in self-regulation which is a predisposition to regulate others (Ham and Tronick, 2009).

In fact, a recent systematic meta-analysis (Cardoso et al., 2014) revealed that the modulatory effect of OT depends both on the specific stressor and the clinical load of the sample. It has been discussed before that individuals with clinical symptoms might benefit most from OT application in terms of reduced cortisol levels (Bartz et al., 2011; Simeon et al., 2011). Some studies showed that subjective stress response and cortisol levels were affected by OT only in those with poor individual coping and emotion regulation abilities, respectively, but not in individuals with adequate coping or emotion regulation abilities (Quirin et al., 2011; Cardoso et al., 2012). In addition, OT effects on cortisol seem to depend on the social context of the stressor (Heinrichs et al., 2003; Eckstein et al., 2014).

There is evidence from animal and human studies that stressful events themselves can trigger endogenous OT release (de Jong et al., 2015). It has also been reported that acute stress stimulates OT secretion in specific subsamples with averse childhood experiences and depending on attachment style (Pierrehumbert et al., 2012; Seltzer et al., 2014), whereas other studies failed to find OT responses to psychosocial stressor (Light et al., 2005; Ditzen et al., 2007).

### Couple Behavior

The influence of OT/placebo administration or dyadic coping on couple behavior during the conflict did not reach statistical significance, which might be due to high scores of positive behavior in general in our sample of healthy couples reporting high relationship satisfaction (ceiling effect). In addition the failure to reach significance might also be due to a lack in statistical power. We would assume that the effect might become significant in a larger or more heterogeneous sample.

To our knowledge, this is the first study to investigate inter-individual stress-management traits in their interaction with OT. One theoretical explanation of our findings is that an individual’s history of relationships and attachment has a modulating influence on the endogenous OT system (Macdonald, 2013). This notion is supported by studies showing changes in endogenous baseline levels or OT release in response to stress after childhood trauma or early life stress (Heim et al., 2009; Opacka-Juffry and Mohiyeddini, 2012).

The involvement of OT in stress regulation and social interaction makes this system a highly relevant candidate to investigate psychobiological mechanisms of social relationships. Thus, in adult couples, dyadic stress coping is a central concept to be related to OT research. The endogenous release of OT, e.g., by intimacy or other forms of positive social interaction has probably stress regulating effects. While the present study used external given OT, these might likewise be true for endogenous released OT, e.g., after intimacy, stressors or breastfeeding (Grewen et al., 2010; de Jong et al., 2015). Indeed, we would assume better stress regulation after activities that trigger OT release in individuals with good dyadic coping skills. In previous studies by our group, intimacy between couples, operationalized as physical affection, was significantly associated with reduced salivary cortisol levels in controlled laboratory experiments and in everyday life (Ditzen et al., 2007, 2008). Clinical applications should take into account these mechanisms, e.g., in couple therapy, but also stress coping trainings might promote these behaviors by including romantic partners or the family.

The study has some limitations. The sample consisted of healthy young couples reporting high relationship satisfaction. Given opposing effects of OT in clinical samples (Bartz et al., 2010), we cannot extrapolate our findings to severe marital problems or to patient populations [see for instance a study in couple with substance abuse by Flanagan et al. (2018)]. Likewise, our sample is restricted to heterosexual couples. Since the OT system seems to react differently in homosexuals (Thienel et al., 2014) and the fact that same-sex couples experience specific stressors to deal with (Randall et al., 2017), it would be of great interest to investigate OT and dyadic coping in same-sex couples. Another relevant factor modulating couple behavior is gender (e.g., Gottman, 1994). In our data, however, there was no significant main or interactional effect of gender on couple behavior and cortisol. Furthermore, our findings may not be applicable for some cultures or couple circumstances, therefore generalizability is limited. Culturally based gender role expectations regarding the role of partners in supporting each other’s’ coping and providing emotional support could moderate how OT or DC affects stress reactivity or couple behavior. This should be investigated in future studies.

Our results in couples – with the intimate partner usually serving as the most relevant social interaction partner for adults – might also have relevance for other central social relationships and types of social interactions in adult life, namely parent-child interactions. Up to 70% of the first-time parents report a decrease in relationship satisfaction after becoming parents (Gottman and Notarius, 2000) and increased occurrence of conflicts, often with child related content (Kluwer and Johnson, 2007). Thus, functional conflict strategies and problem solving capacities are of particular importance, not only for the couple, but also for the developing parent-infant-relationship (Krishnakumar and Buehler, 2000) and subsequently for infant and child development (Zemp et al., 2016). In regards to neurobiological mediators, recent studies showed an association between parental behavior and endogenous OT activity (for an overview, see Zietlow et al., 2016; Feldman, 2017) in mothers, but also in fathers (Naber et al., 2010; Weisman et al., 2014), which could indicate a joint neuroendocrine basis. Therefore, in future studies possible parallels between dyadic stress regulation in the couple and parent-infant-interaction and parenting behavior should be investigated.

## Conclusion

Taken together the presented data suggests that the OT system in interaction with dyadic coping can improve stress regulation. In line with previous studies, we showed that rather than acting stress-regulating *per se*, the neuroendocrine mediator OT depend on individual factors such as the individual’s dyadic coping mechanisms.

Both our empirical results as well as theoretical assumptions underline the importance to investigate real-time social interaction behavior in relation to psychobiological dynamics. Possible implications can be drawn for couple therapy and conflict management, but also help understanding stress coping trainings including romantic partners or the family.

## Author Contributions

A-LZ and ME contributed to analysis and interpretation of data, drafted the manuscript, and approved the final version. CH contributed to analysis and interpretation of data and approved the final version. MH, MS, and GB contributed to the study and manuscript conception and approval of the final version. NN and CR contributed to drafting of the manuscript and approval of the final version. BD contributed to study conception and design, acquisition, analysis, and interpretation of the data, drafting of the manuscript, and approval of the final version.

## Conflict of Interest Statement

The authors declare that the research was conducted in the absence of any commercial or financial relationships that could be construed as a potential conflict of interest. The reviewer JG and handling Editor declared their shared affiliation at the time of the review.
